# Supplementary material for: The genome sequence of Cynthia’s Fritillary, Euphydryas cynthia (Dennis & Schiffermüller), 1775 (Lepidoptera: Nymphalidae)
Source: Wellcome Open Res. 2025 Aug 15;10:447. [Version 1] doi: 10.12688/wellcomeopenres.24696.1 (PMC12648028; doi:10.12688/wellcomeopenres.24696.1)
Supplement: Peer review history

# Reviewer report: Reviewer response for version 1

**DOI:** 10.21956/wellcomeopenres.27212.r139794

**Published:** 2025-12-29

**Authors:** Somjit Homchan

**Affiliations:** 1 Naresuan University, Tha Pho, Thailand

This manuscript is well-prepared, and the protocols employed in this study are robust, providing a comprehensive report of the whole genome sequence. I have a few suggestions to further strengthen the manuscript for Indexing.

 1. Species taxonomy: Please check species, *Euphydryas cynthia* (Dennis & Schiffermüller, 1775), or *Euphydryas cynthia* (Denis & Schiffermüller, 1775). Additionally, I found that the species name is sometimes cited with only one authorship as *Euphydryas cynthia* (Schiffermüller, 1775). Therefore, which one is the right one

 2. Regarding the two haplotypes reported in this genome sequencing, haplotype 1 and haplotype 2 show significant differences. Please clarify for the reader how these differences may have arisen.

 3. The author reported the heterozygosity rate of 1.09%. Is this level typical for this butterfly species? is it low or high? It would be beneficial to include a brief discussion and explain about this value.

Are sufficient details of methods and materials provided to allow replication by others?

Yes

Is the rationale for creating the dataset(s) clearly described?

Yes

Are the datasets clearly presented in a useable and accessible format?

Yes

Are the protocols appropriate and is the work technically sound?

Yes

Reviewer Expertise:

Molecular genetic markers, Population genetics, Cytogenetics

I confirm that I have read this submission and believe that I have an appropriate level of expertise to confirm that it is of an acceptable scientific standard.

# Reviewer report: Reviewer response for version 1

**DOI:** 10.21956/wellcomeopenres.27212.r139790

**Published:** 2025-11-25

**Authors:** Fedor Čiampor

**Affiliations:** 1 Slovak Academy of Sciences, Bratislava, Slovakia

I am not an expert on Lepidoptera taxonomy, but according to gbif.org, *Euphydryas cynthia* (Denis & Schiffermüller), 1775 is a synonym of the species *Hypodryas cynthia* (Denis & Schiffermüller), 1775, but in the European butterfly dataset, which is also indexed on gbif.org, the species is listed in the genus Euphydryas. I cannot judge which is correct, but before the article is indexed, it definitely needs to be verified, as this is basic information about sequenced species. It seems to me that Higgins 1978 described a new genus *Hypodryas* and moved the species *E.cynthia* into it. Perhaps this is potentially incorrect and lepidopterists adhere to the original classification in the genus *Euphydryas*, but no one has formally corrected the taxonomy, so the correct name to use should be *Hypodryas cynthia*. However, maybe the synonymization of *Hypodryas* with *Euphydryas* has been indexed, but I have not found this information. It needs to be found and the currently valid name used. I have received a reaction to my request from the gbif: „GBIF will soon update the taxonomic backbone to the extended release of Catalogue of Life. According to this resource, *Euphydryas cynthia (Denis & Schiffermüller, 1775)* is a synonym of *Hypodryas cynthia ([Denis & Schiffermüller], 1775)*.”

 If invalid/incorrect names are used, this causes serious problems in the use of data. In addition, you list the authors as Dennis & Schiffermüller, but the correct is Denis & Schiffermüller. Furthermore, in the taxonomy of the species, you refer to (NCBI:txid104506), where *Euphydryas cynthia* is listed with incorrect authorship: (Schiffermuller, 1775). I would recommend not refer to NCBI in taxonomy because it is not a very reliable database from a taxonomic point of view.

 I found information about isolated occurrences of the species in Belarus, Montenegro, and Spain. It is good to follow current information about distribution

 The genome of a related butterfly (Coenonympha arcania) from the same family was recently indexed. The authors should comment on whether it is OK that there is such a significant difference in the size of their genomes and the number of chromosomes. What could be the cause of this? This would be helpful for the future use of such generated data.

Are sufficient details of methods and materials provided to allow replication by others?

Yes

Is the rationale for creating the dataset(s) clearly described?

Yes

Are the datasets clearly presented in a useable and accessible format?

Partly

Are the protocols appropriate and is the work technically sound?

Yes

Reviewer Expertise:

invertebrate taxonomy, phylogeny, DNA (meta)barcoding

I confirm that I have read this submission and believe that I have an appropriate level of expertise to confirm that it is of an acceptable scientific standard, however I have significant reservations, as outlined above.

# Reviewer report: Reviewer response for version 1

**DOI:** 10.21956/wellcomeopenres.27212.r139791

**Published:** 2025-11-25

**Authors:** Kristina Gagalova

**Affiliations:** 1 Curtin University, Perth, Australia

The work is well developed and presented. I only have a few comments

 Major

 * What do you think is the difference in size between the two haplotypes, 50Mbp? This may suggest biological and sequencing issues, could you elaborate?

 are the following relevant to explain the discrepancy: repetitive element expansion, hemizygous W regions, assembly overphasing, or uncollapsed haplotigs

 * 1.09% heterozygosity was assumed for the whole genome, is there any difference between the sex and somatic chromosomes? Clarify whether heterozygosity was recalculated after W chromosome filtering and whether any significant difference may be expected

 * Sex chromosomes have a quite different features from somatic chromosomes, can you please separate these from the rest of the results and mention how these contigs were identified and curated, separately from the rest? That will be of value for others assembling similar genomes

 * BlobToolKit was use but there is no description whether biological contaminants are found. Can you please elaborate on this? low-coverage contaminants usually exist, especially with whole-body DNA extractions from insects. How much was found of these and were they removed?

 Minor

 * Which FastK, GenomeScope settings were used

 * Specific Hifiasm parameters

 * Hi-C read processing and filtering thresholds

 * Manual curation criteria (i.e. 8 breaks and 60 joins, why?)

 * Versioning of Nextflow/conda profiles used in pipelines.

 * Is the mitochondrion genome circular?

Are sufficient details of methods and materials provided to allow replication by others?

Yes

Is the rationale for creating the dataset(s) clearly described?

Yes

Are the datasets clearly presented in a useable and accessible format?

Yes

Are the protocols appropriate and is the work technically sound?

Yes

Reviewer Expertise:

genome assemblies, genomics, plant and insects genomes, complex genomes, contamination screening, pangenomes, genome annotations, nextflow pipelines development, HPC workflows

I confirm that I have read this submission and believe that I have an appropriate level of expertise to confirm that it is of an acceptable scientific standard.

# Reviewer report: Reviewer response for version 1

**DOI:** 10.21956/wellcomeopenres.27212.r130721

**Published:** 2025-09-05

**Authors:** Yang Mei

**Affiliations:** 1 Jilin Agricultural University (Ringgold ID: 85112), Changchun, Jilin, China; 2 Zhejiang University, Zhejiang, China

The authors provide a high-quality assembly of *Euphydryas cynthia*, especially including the W chromosome. The methods and results were described well and clearly. I think it could be indexed in the current version.

 But I also wonder why there is 50 mb difference between the two haplotypes. It is because of the high heterozygosity?

 Why is the heterozygosity high based on the one adult sample?

Are sufficient details of methods and materials provided to allow replication by others?

Yes

Is the rationale for creating the dataset(s) clearly described?

Yes

Are the datasets clearly presented in a useable and accessible format?

Yes

Are the protocols appropriate and is the work technically sound?

Yes

Reviewer Expertise:

Insect genomics

I confirm that I have read this submission and believe that I have an appropriate level of expertise to confirm that it is of an acceptable scientific standard.
